# Perceived and objective availability of green and blue spaces and quality of life in people with dementia: results from the IDEAL programme

**DOI:** 10.1007/s00127-021-02030-y

**Published:** 2021-01-23

**Authors:** Yu-Tzu Wu, Linda Clare, Ian Rees Jones, Sharon M. Nelis, Catherine Quinn, Anthony Martyr, Christina R. Victor, Ruth A. Lamont, Isla Rippon, Fiona E. Matthews, The Improving the Experience of Dementia, Enhancing Active Life (IDEAL) Programme

**Affiliations:** 1 Social Epidemiology Research Group, Health Service and Population Research Department, Institute of Psychiatry, Psychology and Neuroscience, King’s College London, London, SE5 8AF UK; 2 College of Medicine and Health, University of Exeter, St Luke’s Campus, Exeter, EX1 2LU UK; 3 Wales Institute of Social and Economic Research and Data, Cardiff University, Cardiff, CF10 3BB UK; 4 Centre for Applied Dementia Studies, University of Bradford, Bradford, BD7 1DP UK; 5 Department of Clinical Sciences, College of Health and Life Sciences, Brunel University London, Uxbridge, UB8 3PH UK; 6 Population Health Sciences Institute, Newcastle University, The Baddiley-Clark Building, Richardson Road, Newcastle upon Tyne, NE4 5PL UK

**Keywords:** People with dementia, Quality of life, Green spaces, Blue spaces, Perceived and objective environment measures

## Abstract

**Purpose:**

The aim of this study was to investigate the associations between quality of life and both perceived and objective availability of local green and blue spaces in people with dementia, including potential variation across rural/urban settings and those with/without opportunities to go outdoors.

**Methods:**

This study was based on 1540 community-dwelling people with dementia in the Improving the experience of Dementia and Enhancing Active Life (IDEAL) programme. Quality of life was measured by the Quality of Life in Alzheimer’s Disease (QoL-AD) scale. A list of 12 types of green and blue spaces was used to measure perceived availability while objective availability was estimated using geographic information system data. Regression modelling was employed to investigate the associations of quality of life with perceived and objective availability of green and blue spaces, adjusting for individual factors and deprivation level. Interaction terms with rural/urban areas or opportunities to go outdoors were fitted to test whether the associations differed across these subgroups.

**Results:**

Higher QoL-AD scores were associated with higher perceived availability of local green and blue spaces (0.82; 95% CI 0.06, 1.58) but not objective availability. The positive association between perceived availability and quality of life was stronger for urban (1.50; 95% CI 0.52, 2.48) than rural residents but did not differ between participants with and without opportunities to go outdoors.

**Conclusions:**

Only perceived availability was related to quality of life in people with dementia. Future research may investigate how people with dementia utilise green and blue spaces and improve dementia-friendliness of these spaces.

## Introduction

Interaction with the natural environment, such as visiting gardens, parks, woodlands and rivers and coastal areas, termed green and blue spaces, has been associated with better health and wellbeing in the general population [1, 2] as well as among people with dementia [3–6]. Previous research has suggested that access to nature might have beneficial influences on physical activity, psychological health and wellbeing in people with dementia and contribute to reduction in dementia-related symptoms [6–10]. However, most existing studies are based on relatively small numbers of people with dementia living in residential care settings and have generally used qualitative methods [3]. There is a lack of empirical evidence from community-dwelling people with dementia and how their interactions with the local natural environment may support people to live well with the condition.

Perceptions of the natural environment are likely to be linked to experiences of environmental interactions in the wider context of social networks, mobility and sociocultural systems [11]. Individual factors, such as sociodemographic characteristics and health status, as well as collective factors, such as values, norms and social networks, may modify behaviour and the use of the environment in certain subgroups [11, 12]. For example, people with low socioeconomic status generally have poor access to green space and the quality of green space is reported to be worse in more deprived areas [13, 14]. People with dementia who experience changes in cognitive function and health status might also have different perceptions of the availability of green and blue spaces, reflecting their experiences of access to and engagement with these environmental features [3]. Thus, perceived availability of green and blue spaces should not be considered as a proxy for objective availability of green and blue spaces, which is generally measured using national statistics, maps or geographic information system (GIS) data [15]. While the objective measures indicate actual areas of green and blue spaces, the perceived measures may reflect individual subjective experience of interacting with these spaces. To investigate the role of green and blue spaces in supporting people to live well with dementia, it is important to consider variation between perceived and objective measures and investigate the potential impact of these on quality of life, a key outcome measure in dementia care research focusing on individual perception of wellbeing, happiness, goodness and satisfaction with various aspects of life [16, 17].

Based on a large cohort study of people with dementia in Great Britain, the aim of this study was to examine perceived and objective availability of local green and blue spaces and their associations with quality of life. The analysis also investigated whether the associations of quality of life with perceived and objective availability of green and blue spaces varied across those with different individual (with and without opportunities to go outdoors) and area-level (urban and rural settings) factors.

## Methods

### Study population

The Improving the experience of Dementia and Enhancing Active Life (IDEAL) programme is a longitudinal cohort study of 1540 community-dwelling people with dementia and 1278 carers in Great Britain [18, 19]. More detailed information on study design and the profile of the IDEAL study population has been reported previously [18, 20]. In brief, IDEAL aims to identify social, psychological and economic factors that support people to live well with dementia. Recruitment was carried out through a network of 29 National Health Service sites across England, Scotland and Wales between July 2014 and August 2016. All participants were required to have a clinical diagnosis of dementia and a Mini-Mental State Examination score ≥ 15 on entry to the study. People with dementia who were not able to provide informed consent were excluded from recruitment. For each person with dementia, a carer who provided practical or emotional unpaid support was also recruited where possible. For those who agreed to take part, a trained researcher visited them at home and implemented standardised questionnaires at baseline and two follow-up interviews 12 and 24 months later. Among 3105 people with dementia who were approached by the IDEAL researchers, 378 were ineligible, 1106 declined and 81 withdrew subsequently. The response rate was 56% among eligible people with dementia.

The study was approved by the Wales Research Ethics Committee 5 (reference: 13/WA/0405) and the Ethics Committee of the School of Psychology, Bangor University (reference: 2014-11684). The study is registered with the UK Clinical Research Network, registration number 16593. This analysis focused on the baseline data of 1540 people with dementia.

### Quality of life

People with dementia rated their quality of life using the Quality of Life in Alzheimer’s Disease scale (QoL-AD), a dementia-specific measure of quality of life with good internal consistency (Cronbach’s *α* = 0.88) and test–retest reliability (intra-class correlation coefficient = 0.76) [21]. In the IDEAL study, the Cronbach’s *α* of QoL-AD was estimated to be 0.86 [22]. The scale includes 13 items on mood, memory, functional abilities and interpersonal relationships and a 4-level Likert response scale for each item (poor, fair, good and excellent). A higher score (range 13–52) indicates better quality of life.

### Perceived availability of green and blue spaces

To measure the availability of local green and blue spaces, the IDEAL questionnaire provided a list of different possible types of green and blue space and people with dementia were asked which of the following 12 types of green and blue spaces were within a 10-minute walk of their dwelling (yes/no): countryside; woodlands; parks and gardens; country parks; green corridors; outdoor sports facilities; amenity green space; play areas; allotments, community gardens and urban farms; cemeteries and churchyards; river, lake or canal; and sea [23]. The option of ‘none of the above’ was also provided. The total number of local green and blue spaces was calculated based on the sum of these 12 items and was divided into quartiles (0–4, 5–6, 7–8, 9–12). A higher number indicates perceived availability of more of these spaces in the local area.

### Objective availability of green and blue spaces

GIS data were used to measure objective availability of green and blue spaces in local areas. The Ordnance Survey (OS) Open Greenspace data [24] provide the location and extent of green spaces that are likely to be accessible to the public across Great Britain, including allotments or community growing spaces, bowling greens, cemeteries, religious grounds, golf courses, other sports facilities, play spaces, playing fields, public parks or gardens, and tennis courts. The OS Open Rivers data [25] provide information on the watercourse network including freshwater rivers, tidal estuaries and canals in Great Britain. Given the time points of the IDEAL baseline interview, the earliest available versions were obtained for the greenspace (version July 2017) and river data (version October 2016) via Digimap (digimap.edina.ac.uk).

Postcode information relating to the IDEAL participants’ places of residence was converted into the National Grid reference, a UK-based coordinate system [26]. For each participant, a 400-metre (m) buffer based on individual residence was generated as a proxy for a 10-min walking area for people with dementia [27]. The areas (square metres) of green spaces within the buffers were calculated and divided into quartiles. To match with the perceived measure, different types of green spaces were categorised into five groups: allotment or community growing spaces; cemeteries or religious grounds; public parks or gardens; sports spaces; and play spaces. For blue spaces, the OS Open Rivers data were used to identify any rivers within the 400-m buffers and the UK coastline was used to identify seas within the buffers. The measure for blue spaces was categorised into three groups: none; river or sea; and both. All GIS data were managed and analysed using ArcGIS 10.3.1.

### Covariates

The IDEAL interviews collected information on age, sex, education and social class for people with dementia. The highest educational qualification was used to divide participants into three groups: low (no qualification), middle (school-leaving qualification at age 16) and high (higher school-leaving qualification at age 18 or above). Social class was defined using the main occupation in working life and the current standard occupational classification for the UK (SOC 2010) system. The measure was categorised into three social class groups: high (professional or managerial occupations); middle (skilled occupations); and low (partly skilled, unskilled occupations or armed forces). In addition to sociodemographic factors, health status and walking speed might affect perceived availability of local green and blue spaces. Self-rated health over the past 4 weeks was used to measure overall health status and categorised into three groups: very poor/poor; fair; good/very good/excellent [28]. Self-reported walking speed of people with dementia was divided into three levels: slow pace; steady average pace; brisk or fast pace [29]. The opportunity to go outdoors in the last 2 weeks was measured using a single question: “Have you had an opportunity to be outside, go for walks, enjoy nature?” with a binary answer of yes/no [30].

Two area-level measures, area deprivation and rural/urban areas, were linked to the IDEAL participants through postcode information. More detailed information is provided in a previous study [31]. In brief, the deprivation index, which summarised characteristics related to poverty and socioeconomic disadvantage including income, employment, education and training, health and disability, barriers to housing and services, the living environment and crime, were divided into quintiles among all area units for England, Scotland and Wales. The first quintile (Q1) represents 20% of the most deprived areas in the country. Rural/urban areas were defined based on 2011 Census Rural Urban Classification (England and Wales) and Scottish Government Urban Rural Classification 2013–2014 (Scotland).

### Statistical analyses

To investigate whether the perceived availability of local green and blue spaces corresponded to the objective measures, the percentages of agreement (both available or not available) were reported for specific types of green and blue spaces, including parks, sport spaces, play areas, allotments and community gardens, cemeteries and religious grounds, rivers and seas. Since a 10-min walking distance may differ due to individual walking speed, the percentages of agreement were also stratified by self-reported walking speed.

Regression modelling was carried out to investigate the associations of quality of life with perceived and objective availability of green and blue spaces in people with dementia. The quartiles of perceived and objective availability measures were fitted in the modelling and adjusted for sociodemographic factors (age, gender, education and social class), self-rated health, walking speed and area deprivation. A full model was fitted to include both perceived and objective measures in order to test whether one of them had stronger associations with quality of life. Multicollinearity was checked using variance inflation factor (VIF) and remained low in the full model (VIF = 1.60). Since people who had opportunities to go outdoors might have different interactions with green and blue spaces from those who did not have these opportunities, the analysis included interaction terms between opportunities to go outdoors (yes/no) and perceived or objective measures for green and blue spaces. Green and blue spaces in urban areas may ameliorate noise, heavy traffic and pollution and therefore could be particularly important to urban residents. To investigate whether the associations of quality of life with green and blue spaces differed across urban and rural settings, interaction terms between rural/urban areas and perceived or objective measures for green and blue spaces were fitted into the modelling. Robust standard errors were estimated for all models.

To address missing data, multiple imputation was conducted using all variables included in the modelling. Estimates from 20 imputed datasets were combined using Rubin’s rules [32]. The imputed results of regression modelling are reported in this study. Test for trend was used to examine whether higher availability of local green and blue spaces was associated with higher quality of life. This study was based on the IDEAL baseline data version 4.5. All statistical analyses were conducted using Stata 15.1.

## Results

Among the 1540 participants, the median age was 77 years with an interquartile range between 71 and 83. The percentage of men was 56.2% (Table 1). Most participants had secondary education or above (54.1%), professional or managerial occupations (44.7%) and lived in the least deprived areas (30.5%). Nearly 63% reported good, very good or excellent health. There were 46.1% people with dementia reporting average walking speed and 16.5% did not have opportunities to be outside and enjoy nature. Just over two-thirds of participants lived in urban settings.

**Table 1:** Descriptive information about the study population (*N* = 1540)

Individual factors	*N* (%)	Green/blue spaces	*N* (%)
Age		Perceived availability of green/blue spaces within a 10-min walk (missing = 49)	
< 65	134 (8.7)	Countryside	1022 (68.5)
65–69	177 (11.5)	Woodlands	830 (55.7)
70–74	258 (16.8)	Parks and gardens	1038 (69.6)
75–79	368 (23.9)	Country parks	322 (21.6)
80+	603 (39.2)	Green corridors (e.g., river banks or roadside grass verges)	971 (65.1)
Sex		Outdoor sports facilities	668 (44.8)
Men	866 (56.2)	Amenity green space (e.g., public playing fields or football pitches)	902 (60.5)
Women	674 (43.8)	Play areas	919 (61.6)
Education (missing = 35)		Allotments, community gardens, urban farms	599 (40.2)
Low	421 (28.0)	Cemeteries and churchyards	895 (60.0)
Middle	269 (17.9)	River, lake or canal	677 (45.4)
High	815 (54.1)	Sea	182 (12.2)
Social class (missing = 78)		None of above	20 (1.3)
Low	205 (14.0)	Objective availability of green/blue spaces within a buffer of 400 m	
Middle	604 (41.3)	Parks	325 (21.1)
High	653 (44.7)	Sport spaces	613 (39.8)
Self-rated health (missing = 5)		Play spaces and fields	990 (64.3)
Good/very good/excellent	966 (62.9)	Allotments and community gardens	379 (24.6)
Fair	362 (23.6)	Cemeteries and religious grounds	699 (45.4)
Very poor/poor	207 (13.5)	River	523 (34.0)
Walking speed (missing = 45)		Sea	68 (4.4)
Slow pace	530 (35.5)	Urban/rural	
Steady average pace	690 (46.1)	Urban	1036 (67.3)
Brisk/fast pace	275 (18.4)	Rural	504 (32.7)
Opportunity to go outdoors (missing = 11)		Area deprivation	
Yes	1277 (83.5)	Q5 (least)	469 (30.5)
No	252 (16.5)	Q4	380 (24.7)
		Q3	327 (21.2)
		Q2	234 (15.2)
		Q1 (most)	130 (8.4)

Table 2 shows percentages of agreement between perceived and objective availability of green and blue spaces. Apart from sea (90.4%), the agreement was low in all types of green and blue spaces with the percentages ranging between 44.7 and 64.4%. The percentages of agreement remained similar when stratified by walking speed.

**Table 2:** Percentage of agreement between perceived and objective availability of green and blue spaces (%)

Perceived–objective 400 m	Overall	Self-rated walking speed		
		Slow pace	Steady average pace	Brisk/fast pace
Parks	44.7	47.2	42.0	46.3
Sport spaces	57.1	59.9	52.9	61.7
Play spaces and fields	60.3	63.4	58.6	57.6
Allotments and community gardens	64.4	65.7	64.1	62.8
Cemeteries and religious grounds	58.6	61.3	56.8	58.4
River	62.0	64.0	60.2	63.1
Sea	90.4	90.6	90.5	89.8

The mean QoL-AD score was 36.8 with a standard deviation (SD) of 5.9 among the 1396 participants without missing items. Table 3 reports the associations between quality of life and both perceived and objective availability of local green and blue spaces. Higher QoL-AD scorers were found for those reporting a higher availability of perceived green and blue spaces (1.31; 95% CI 0.46, 2.16) but not for those where objective measures of green and blue spaces indicated higher availability. After adjusting for all covariates, the association between quality of life and the perceived availability of green and blue spaces was attenuated (0.82; 95% CI 0.06, 1.58) but the test for trend remained statistically significant. The effect sizes were similar when including both perceived and objective measures for green and blue spaces in one model.

**Table 3:** The associations between quality of life, perceived and objective availability of green and blue spaces

	Model 1	Model 2	Model 3	Model 4
	Coeff. (95% CI)	Coeff. (95% CI)	Coeff. (95% CI)	Coeff. (95% CI)
Perceived number of green/blue spaces within a 10-min walk				
Q1	Ref	Ref	Ref	Ref
Q2	0.40 (− 0.43, 1.22)	0.41 (− 0.40, 1.24)	0.34 (− 0.35, 1.03)	0.35 (− 0.34, 1.04)
Q3	0.81 (− 0.03, 1.64)	0.85 (0.02, 1.69)	0.81 (0.09, 1.53)	0.85 (0.13, 1.58)
Q4	1.31 (0.46, 2.16)	1.41 (0.54, 2.29)	0.82 (0.06, 1.58)	0.84 (0.08, 1.60)
* p* value	< 0.01	< 0.01	0.01	0.01
*R*^2^	0.7	3.8	29.7	30.0
Objective area of green spaces within 400 m				
Q1	Ref	Ref	Ref	Ref
Q2	− 0.98 (− 1.82, − 0.14)	− 0.79 (− 1.63, 0.05)	− 0.71 (− 1.43, 0.01)	− 0.75 (− 1.47, − 0.32)
Q3	− 0.89 (− 1.72, − 0.06)	− 0.76 (− 1.59, 0.08)	− 0.22 (− 0.92, 0.48)	− 0.27 (− 0.98, 0.43)
Q4	− 1.04 (− 1.88, − 0.20)	− 0.83 (− 1.68, 0.01)	− 0.54 (− 1.28, 0.19)	− 0.60 (− 1.33, 0.14)
* p* value	0.03	0.07	0.34	0.27
*R*^2^	0.5	3.4	29.6	30.0
Objective availability of blue spaces within 400 m				
None	Ref	Ref	Ref	Ref
River or sea	0.37 (− 0.27, 1.00)	0.26 (− 0.36, 0.90)	− 0.10 (− 0.65, 0.45)	− 0.15 (− 0.54, 1.84)
Both	0.72 (− 1.18, 2.62)	0.54 (− 1.37, 2.46)	0.33 (− 1.21, 1.86)	0.23 (− 0.46, 1.64)
* p* value	0.20	0.35	0.91	0.69
*R*^2^	0.1	3.1	29.4	30.0

Model 1: unadjusted; model 2: adjusted for age, gender, education and social class; model 3: adjusted for age, gender, education, social class, self-rated health, walking speed and area deprivation; model 4: included both perceived and objective measures for green and blue spaces and adjusted for age, gender, education, social class, self-rated health, walking speed and area deprivation; *p* value: test for trend

**Fig. 1:**
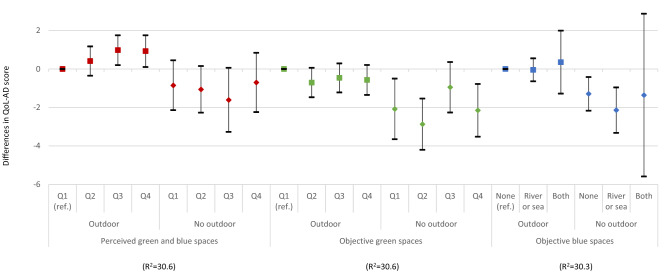
The associations between quality of life, perceived and objective availability of green and blue spaces by participants with and without opportunities to go outdoors (estimated differences in QoL-AD scores and 95% confidence intervals)

**Fig. 2:**
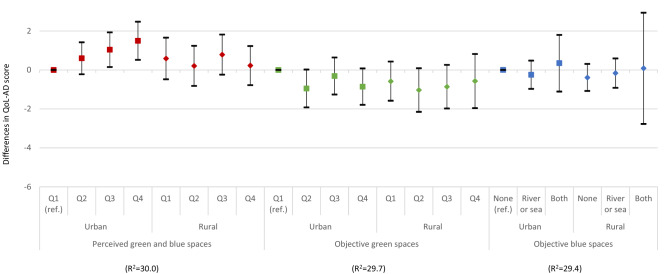
The associations between quality of life, perceived and objective availability of green and blue spaces by urban and rural settings (estimated differences in QoL-AD scores and 95% confidence intervals)

The associations of quality of life with perceived and objective availability of green and blue spaces did not differ across those with and without opportunities to be outside and enjoy nature (Fig. 1). Although people with dementia who did not go outdoors had lower quality of life scores, the interaction terms did not achieve statistical significance for perceived (*p* = 0.24) or objective green (*p* = 0.34) and blue spaces (*p* = 0.36). Urban and rural differences were only found in perceived availability of green and blue spaces (*p* = 0.03) (Fig. 2). In urban areas, a higher availability of perceived green and blue spaces was associated with higher quality of life (1.50; 95% CI 0.52, 2.48) but the association was less clear in rural areas.

## Discussion

### Main findings

Using a large cohort study of people with dementia in Great Britain, this study investigated the associations of quality of life with perceived and objective availability of local green and blue spaces. The agreement between perceived and objective measures for availability of green and blue spaces was generally low. Quality of life in people with dementia was associated with perceived availability of local green and blue spaces but not with objective measures of availability after adjusting for sociodemographic factors, self-rated health, walking speed and area deprivation. High availability of perceived green and blue spaces was particularly important for those living in urban settings.

### Strengths and limitations

This study included a large and geographically spread sample of community-dwelling people with dementia in Great Britain. The GIS data which provide complete information on green and blue spaces across the three countries were integrated with the IDEAL cohort data. In addition to green spaces, the measure also included blue spaces, a different aspect of the natural environment and a novel research focus [33]. Moving beyond previous qualitative research, this analysis used quantitative approaches to compare the potential benefits of both perceived and objective availability of local green and blue spaces for quality of life in people with dementia taking into account individual and area level factors.

Due to the cross-sectional nature of this study, the results cannot indicate causal relationships. People with dementia might have changed their place of residence due to their health conditions and care needs, and hence might have been unfamiliar with new neighbourhood environments. However, 81% of participants had lived in their current place of residence for over five years. The GIS data for the objective availability of green and blue spaces were produced in 2016/2017, which was slightly later than the majority of baseline interviews. Yet areas of green and blue space should have been stable over this short period of time. The study population only included people with mild-to-moderate dementia and therefore the results cannot be generalised to those with severe dementia, who are likely to be homebound and have poor health and/or mobility. This study did not investigate how people with dementia used local green and blue spaces in their daily life. People with dementia might travel to certain green and blue spaces which are outside their local areas. In particular, the objective measures for local green and blue spaces were generated based on the 400-m buffers and this did not take into account individual mobility and activity space. However, the disagreement between perceived and objective measures of availability of green and blue spaces was not explained by walking speed. Participants who reported greater availability were likely to have more frequent interactions with the natural environment in their local areas and therefore the perceived measure might be influenced by level of usage of local green and blue spaces.

### Interpretation of findings

Only perceived availability of local green and blue spaces was associated with quality of life in people with dementia. Low agreement between the perceived and objective availability measures confirmed that these two measures captured different aspects of local green and blue spaces and their relevance for people with dementia. While this study focused on people with mild-to-moderate dementia, the results correspond to the large body of previous quantitative research on healthy adults and older people, which has reported inconsistency between perceived and objective environmental measures and different relationships with physical activity and walking [34–36], mental health [37] and health-related quality of life [38]. This demonstrates that although people with dementia may have memory problems and difficulties with orientation, variation between perceived and objective environmental measures is likely to be observed in both those with and without dementia. On the other hand, evidence from qualitative research generally highlights positive experiences from engagement with nature in older people, including people with dementia [3]. Perceived availability was based on participants’ recollection, and participants might mainly report those green and blue spaces that are relevant to their daily lives. Compared to the objective availability measures, these perceived green and blue spaces might be more ‘meaningful’ due to people with dementia being able to access, use or enjoy these spaces. The different relationships with perceived and objective availability measures might reflect variation in participants’ interactions with nature or differential availability or quality of green and blue spaces which were easily accessible for people with dementia [39].

Perceived availability of local green and blue spaces was particularly important for people with dementia living in urban settings. Environmental stressors in urban areas such as noise, traffic and pollution can lead to distress and sensory overload [40, 41]. People with dementia who reported a higher number of local green and blue spaces might have more frequent interactions with these spaces in urban areas and experience stress reduction and psychological restoration in nature [2, 6]. Opportunities to go outdoors did not modify the associations of quality of life with perceived and objective availability of green and blue spaces. Although people who were homebound might not be able to directly interact with local green and blue spaces, they might still enjoy aesthetic views of nature from widows or balconies and receive some benefits of exposure to greenness [42].

### Implications and future research directions

To enhance the positive influence of green and blue spaces on quality of life, it is important to explore how people with dementia interact with local green and blue spaces and examine their subjective experience of this. Qualitative research has identified several barriers to accessing green spaces [27, 43]. This includes individual factors such as lack of motivation and physical fitness and environmental issues such as safety concerns, accessibility, quality of green spaces and lack of dementia-friendly awareness [27, 39, 43]. Future research may investigate these issues in longitudinal studies, identifying potential changes with the progression of dementia and specific barriers and needs in those with advanced dementia. Recent studies have also used wearable devices to provide accelerometer and Global Position System (GPS) data [11]. This approach may provide empirical data on how people with dementia utilise green and blue spaces and inform possible interventions relating to specific individual and environmental factors.

## Data Availability

IDEAL data were deposited with the UK data archive in April 2020 and will be available to access from April 2023. Details of how the data can be accessed after that date can be found here: https://reshare.ukdataservice.ac.uk/854293/.
